# Central nervous system commitment in Chagas disease

**DOI:** 10.3389/fimmu.2022.975106

**Published:** 2022-11-10

**Authors:** Yerly Useche, Ana Rosa Pérez, Juliana de Meis, Adriana Bonomo, Wilson Savino

**Affiliations:** ^1^ Laboratory on Thymus Research, Oswaldo Cruz Institute, Oswaldo Cruz Foundation, Rio de Janeiro, Brazil; ^2^ Institute of Clinical and Experimental Immunology of Rosario (IDICER CONICET UNR), Rosario, Argentina; ^3^ Center for Research and Production of Biological Reagents (CIPReB), Faculty of Medical Sciences National University of Rosario, Rosario, Argentina; ^4^ National Institute of Science and Technology on Neuroimmunomodulation, Oswaldo Cruz Institute, Oswaldo Cruz Foundation, Rio de Janeiro, Brazil; ^5^ Rio de Janeiro Research Network on Neuroinflammation, Oswaldo Cruz Institute, Oswaldo Cruz Foundation, Rio de Janeiro, Brazil

**Keywords:** Chagas disease, central nervous system, neuroimmune interactions, encephalitis, brain inflammation, oral *Trypanosoma cruzi* transmission

## Abstract

The involvement of the central nervous system (CNS) during human acute and chronic Chagas disease (CD) has been largely reported. Meningoencephalitis is a frequent finding during the acute infection, while during chronic phase the CNS involvement is often accompanied by behavioral and cognitive impairments. In the same vein, several studies have shown that rodents infected with *Trypanosoma cruzi* (*T. cruzi*) display behavior abnormalities, accompanied by brain inflammation, *in situ* production of pro-inflammatory cytokines and parasitism in diverse cerebral areas, with involvement of microglia, macrophages, astrocytes, and neurons. However, the mechanisms used by the parasite to reach the brain remain now largely unknown. Herein we discuss the evidence unravelling the CNS involvement and complexity of neuroimmune interactions that take place in acute and chronic CD. Also, we provide some clues to hypothesize brain infections routes in human and experimental acute CD following oral infection by *T. cruzi*, an infection route that became a major CD related public health issue in Brazil.

## Introduction

Chagas disease (CD) is caused by the protozoan parasite *Trypanosoma cruzi* (*T. cruzi*) and affects approximately 6–8 million people, being endemic in 21 countries from the south of the United States to southern Argentina and Chile. Migration of infected people has spread the disease in non-endemic regions in Europe, North America, Asia, and Oceania (1). In human CD, around 20-30% of chronically infected patients develop cardiac and/or gastrointestinal damage, with cardiovascular disease-induced early mortality and loss of productivity. These data illustrate to what extent CD remains a public health issue, also providing an economic argument for the need of urgent efforts towards controlling CD (2, 3).

Transmission of CD was classically described as being dependent on the insect vector bite. Nowadays, infection through the oral route had gained attention, while transmission through an insect bite, placenta, transfusions, and transplants have been better controlled (4). In the period between 1968-2000, more than 50% of CD acute cases in the Brazilian Amazon region were attributable to micro-epidemics due to orally transmitted infection (5). Moreover, oral infection outbreaks have been reported in several South American countries, being associated with *T. cruzi* presence in food or beverage consumed by the habitants of endemic regions (6). A recent epidemiological study of acute CD indicates a total of 5,184 cases recorded with an annual incidence rate of 0.16 per 100,000 habitants/year. This study reported an increased frequency of oral transmission in acute cases in the North Brazilian region, from 493 cases in the first period from 2005-2009 to 1770 cases in the second period 2010-2018, thus increasing almost 30% in the north of Brazil (7).

The involvement of the CNS during human acute and chronic phases of CD has been reported. Meningoencephalitis has been described more frequently in children during the acute disease, while in the chronic phase neuritis is related to sensory impairment reported in up to 10% of patients. Dementia, confusion, chronic encephalopathy, and sensitive and motor deficits are less frequent. Moreover, experimental infections with several parasite strains demonstrated behavioral alterations (8–10). In line with these evidences, HIV and transplant patients, both immunesuppressed, often show parasite reactivation with meningoencephalitis and brain abscesses (11). In this regard, experimental model of oral acute infection cleary showed the presence of parasite in brain regions a few days after infection (12).

Herein we will focus on the neuroimmune interactions in acute and chronic CD, mostly studied following vector infection route. Moreover, we provide some clues concerning posible CNS changes in orally transmitted *T. cruzi* in experimental infections.

## Clinical CNS manifestations in human Chagas disease

The involvement of the CNS during *T. cruzi* infection in both acute and chronic phases has been reported decades ago. Encephalitis may occur during the acute phase, accompanied by elevated levels of albumin, leukocytes, and trypomastigotes in the cerebral spinal fluid -CSF- (13). Also, acutely infected patients manifest difficulty in mental concentration, cephalea, muscular disturbances (myoclonus, bradykinesia, dyspraxia), weakness, and speech disturbances (14). Headaches, seizures, lethargy, or mood changes are probably due to meningoencephalitis, exhibited by 5-10% of *T. cruzi* acutely infected patients (11). This condition more frequently affects children under 2 years of age and is almost always fatal when coexists with myocarditis and cardiac insufficiency (15). Of note, the acute congenital phase may be associated with seizures and meningoencephalitis in a small proportion of children (16).

Chronically infected patients with dementia, confusion, as well as sensorial and motor deficits occur, have been reported. Additionally, motor deficits associated with neuritis result in altered tendon reflexes and sensory impairment (11). Decreased orientation, attention, and cognitive performance (i.e. lower Mini-Mental State Exam scores) were reported in chronic CD patients compared with healthy controls. Moreover, lower scores of the Weschler Memory Scale and the WAIS global test of Intelligence were associated with chronic CD. The cognitive dysfunction is compatible with signs characteristic of White Matter Disease, with impairment in non-verbal reasoning, information processing, problem-solving and learning (17).

It is noteworthy that CNS is the most frequent site of *T. cruzi* reactivation in immunosuppressed HIV-infected patients (18). Also, the reactivation in this situation is accompanied by brain tissue damage related to increased IL-17 expression (19), as well as low peripheral blood CD4 T-cell counts (11). Of note, meningoencephalitis usually coincides with abundant trypomastigotes in the CSF (20).

We should also point out that proinflammatory cytokines, as well as pathogen associated molecular patterns (LPS and poli:IC) induce depressive behavior (21). Accordingly, during CNS infection by *T. cruzi*, macrophages, microglia and astrocytes can release TNF-α, IL-1β, and nitric oxide -NO- (22, 23), which seems to be related to neurological alterations observed in CD. Also, glutamate release is a common result of CNS infection, being induced by TNF-α and LPS (24). These data should be placed in the context that glutamate is the most prevalent excitatory neurotransmitter in the CNS and plays a role in basic brain capacities, being altered in several psychiatric and neurological disorders (25).

## Changes in the CNS following experimental *T. cruzi* infection correlate with data seen in patients with Chagas disease

Experimental evidence showed CNS alterations and parasite presence in the brain after *T. cruzi* infection, despite the entry routes (**Table 1**). Frequently, CNS infection occurs during the acute infections around the peak of parasitemia, although parasite load and number of inflammatory *foci* are more conspicuos in the basal ganglia when parasitemia is no longer detectable (32). Interestingly, amastigote nests appear in the neuropil of a cerebellar gray matter nucleus, in the absence of a detectable inflammatory process (28). Actually, diverse brain regions can be parasitized by *T. cruzi*. Concurrent to hypothalamic and pituitary detection of *T. cruzi* after infection, several alterations and injury in the hypothalamic–pituitary–adrenal (HPA) axis were reported, caused either directly by parasite invasion or indirectly as a consequence of local and/or systemic inflammation in response to infection (33–38). Brain regions frequently infected by *T. cruzi*, beside the ones described above, are the meninges, cerebral and cerebellar cortices (28, 29), which leads to meningoencephalitis. Other less frequently infected regions are the hippocampus (27), as well as areas without BBB, such as the choroid plexus (29).

**Table 1:** Experimental *T. cruzi* infection models with CNS involvement.

CNS infected regions/cells	Rodent model	*T. cruzi* strain* (trypomastigotes)	Infection route	References
Brain	C3H/HeJ mice	Tulahuen	Subcutaneous	(26)
Meningoencephalitis, Choroid plexus, Hippocampus	C3H/HeJ mice	Colombian	Intraperitoneal	(27)
Meninges, Cerebral cortex	Swiss mice	Colombian	Intraperitoneal	(10)
Gray and white matter in cerebral and cerebellar cortices, Astrocytes	Holtzman rats	Y, CL, PNM	Intraperitoneal	(28)
Meningoencephalitis, Leptomeninges, Parenchyma, Choroid plexus, Cerebellum	C57BL/6 mice	Colombian	Intraperitoneal	(29)
Olfactory bulb, Pituitary gland	BALB/c mice	Dm28c	Oral	(12)
Parenchyma, Hippocampus, Cerebellum, Astrocytes, Microglia	C3H/HeJ mice C57BL/6 mice	Colombian	Intraperitoneal	(30)
Hypothalamus	Wistar rats	Y	Intraperitoneal	(31)
Basal ganglia, Cortex Cerebellum	C57BL/6 mice	Tulahuen	Intranasal	(32)
Pituitary gland	BALB/c mice	Colombian	Intraperitoneal	(33)

*Corresponding *T. cruzi* discrete typing units (DTUs): DTU-I: Colombian, Dm28c, Tulahuen; DTU-II: SC2005, Y; DTU-VI: CL.

Histological studies of *T. cruzi*-infected rodents showed injured CNS regions with neuronal loss (31), glial nodules (due to astrocyte proliferation), edema, and enlargement of perivascular spaces (27, 31), as well as the appearance of perivascular and intraparenchymal mononuclear cellular infiltrates (10, 18, 27–29, 31).

Experimental infections with Colombian and H4 *T. cruzi* strains also demonstrated behavioral abnormalities, such as sleep and memory deficits, anxiety, and depression (8–10, 30). In the same vein, depressive behavior, probably associated with oxidative stress and enhanced pro-inflammatory cytokines has been recorded in chronic CD patients [see review by (39)]. Colombian strain-infected mice showed significant increased immobility and signs of depression both during the acute and chronic infections. Interestingly, TNF-α inhibitors (i.e. pentoxifylline or anti-TNF), as well as the antidepressant fluoxetine, ameliorate depressive-like alterations in this model (30), suggesting a role for systemically TNF-α release in *T. cruzi*-induced depression.

**Figure 1:**
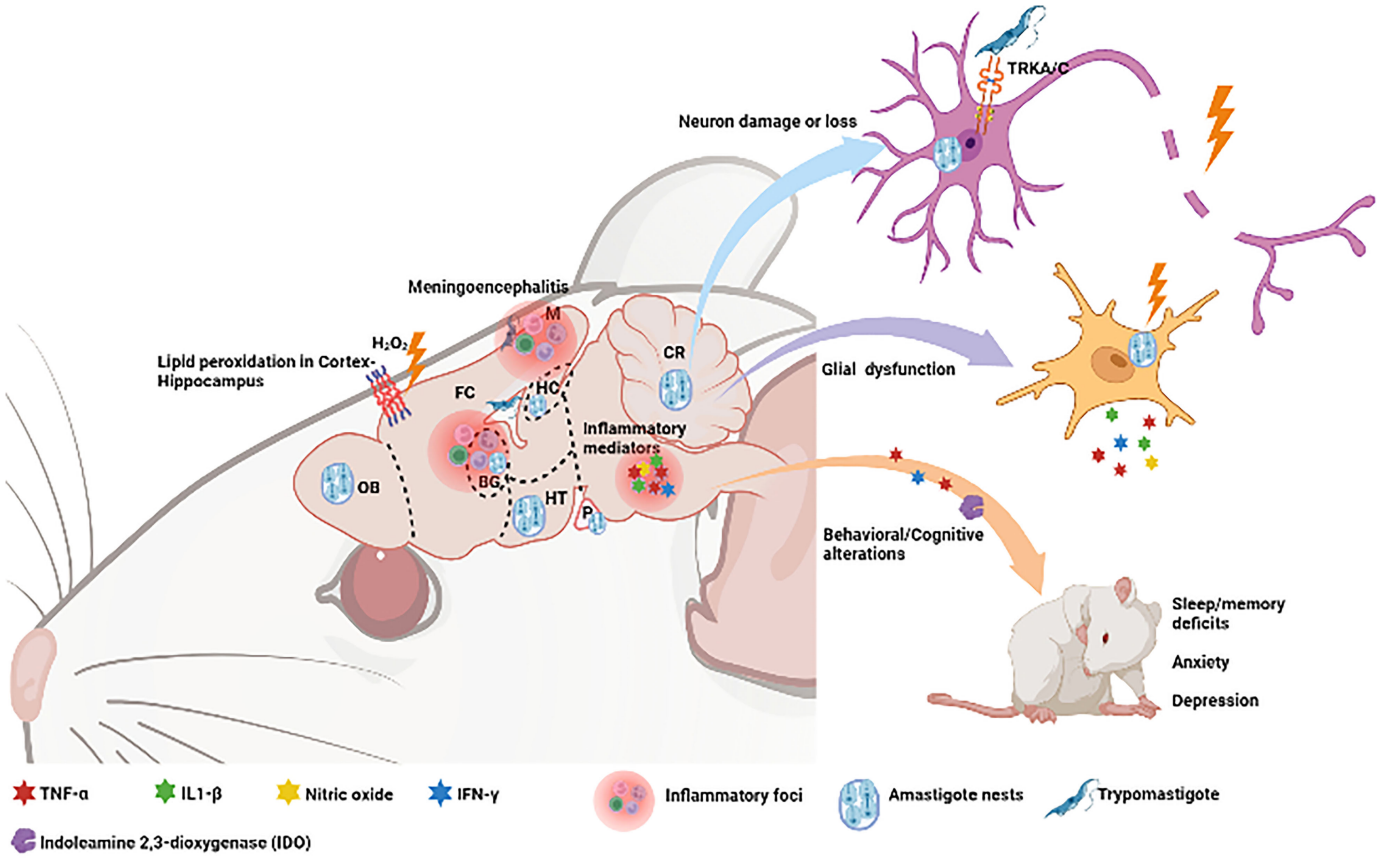
Consequences of experimental *T. cruzi* infection upon the Central Nervous System and respective involved regions. The presence of the parasite in these regions can cause lipid peroxidation as well as neuroinflammation with release of inflammatory mediators, glial dysfunction and neural damage. Secretion of proinflammatory cytokine triggers behavioural and congnitive changes with deficits in sleep and memory, as well as symptoms of anxiety and depression. Olfactory Bulb (OB), Frontal Cortex (FC), Meninges (M), Basal Ganglia (BG), Hippocampus (HC), Hypothalamus (HT), Cerebellum (CR). Neurotrophin receptors (TRKA/C). Created with BioRender.com.

Chronic depressive-like behavior appears to be parasite strain-dependent, since depression was triggered by the Colombian but not by the Y strain. Probably the depressive-like behavior was driven by the increased indoleamine 2,3-dioxygenase (IDO) expression in the CNS in acute and chronic *T. cruzi* infection (30). IDO is a tryptophan-catabolizing enzyme, induced by IFN-γ and TNF-α in infected tissues, including the brain. In addition to depression, chronically infected C57BL/6 mice with the Colombian strain exhibit anxiety and alterations of motor coordination, not associated with sickness behavior signs such as temperature variations or weight loss (9). Other behavioral distubances in chronically *T. cruzi*-infected mice may occur whithout neuroinflammation, including deficits in spatial habituation, novel object recognition, and aversive memory recall. Of note, *T. cruzi* persistence and increased lipid peroxidation in the CNS hippocampus and cortex were associated with these cognitive alterations (40). All these data are summarized in **Figure 1**. Interestingly, in the brain of patients suffering from depression, tryptophan depletion by IDO affects serotonin synthesis and low serotonin levels ocurr, being a possible mechanism related to depression (41).

## *T. cruzi* “strategies” for CNS invasion

As mentioned above, several studies have shown that *T. cruzi* does infect the CNS (12, 26–33). Yet, the mechanisms used by the parasite to reach brain tissues remain largely unknown.

Successful spread from a point of entry to the CNS, either by crossing or disrupting the blood brain barrier (BBB) or the cerebrospinal fluid barrier (BCSFB), involves evasion from both peripheral and intra-CNS immune responses. The BBB and BCSFB are selectively permeable to macromolecules and hydrophilic molecules, due to tight junctions, particularly prominent in the BBB (42). Moreover, leptomeningeal vessels as well as CNS regions bearing BCSFB (i.e. choroid plexus and brain circumventricular organs) are more permeable than vessels in the parenchyma (43). Additionally, the immune response in the meninges is stronger than that seen in the brain parenchyma (42). Also, circulating immune cells, including monocytes, T cells, and macrophages, increase their passage through the brain endothelial cells (BEC) when activated; a process mediated by astrocyte-released cytokines and chemokines, which induce the expression of BEC adhesion molecules in response to infection (44).

In *T. cruzi* infected newborn rats, parasites were detected at the peak of parasitemia inside glial cells, near neuronal somas, capillaries, and venules in the cerebral and cerebellar cortices and the thalamic subcortical nuclei (28). This parasite localization suggests a possible invasion through the BBB vessels, as for example, by the BBB endothelial junction’s impairment mediated by bradykinin, as occurs in cerebral malaria (45). It is known that parasite-derived cruzipain cleaves plasma kininogens into bradykinin, which activates the endothelial bradykinin-B2 receptors and protein-G immune pathway (46). As shown *in vitro*, it is conceivable that *T. cruzi* migrates through a paracellular way across BECs without monolayer disruption, mediated by bradykinin and CCL2 gradient (47).

Increment in plasma TNF-α levels or CNS *Tnf* mRNA expression during murine *T. cruzi* acute infection has been reported. For instance, orally *T. cruzi* infected mice present higher parasitemia, mortality rates, and TNF-α serum levels (48, 49). Interestingly, *Trypanosoma brucei* (*T. brucei*), which is another trypanosomatid parasite, crosses the disrupted BCSFB mediated by TNF-α overexpression in the choroid plexus and circumventricular organs during infection in rodents (50). Actually, *T. brucei* requires the TLR-MyD88 signal mediation to penetrate the brain and generate microglial activation (51).

An increase in metalloproteinases (MMPs) is another inductor factor of BBB dysfunction, as reported in *T. brucei* CNS infection in mice (52). MMPs cleave specific collagen types in the basement membranes and degrade endothelial tight junctions, improving the parasite invasion through the BBB (53). Although the activation of the TLR-MyD88 pathway has not been reported in CNS infection by *T. cruzi*, it is a plausible scenario.

Several areas with BCSFB, as the choroid plexus and hippocampus, presented mild to intense inflammatory infiltrates during experimental acute and chronic *T. cruzi* infection (27, 29). Moreover, amastigote nests were seen in the region between the cerebellum and the spinal cord (cerebellar neuropil) (28), and below the pia mater in the ependymal cells that form the glia limitans at the brain surfaces (10). These findings indicate that *T. cruzi* can cross the endothelium of the leptomeningeal venules in the subarachnoid space, traverse the pia mater till the glia limitans, and gain access to the brain parenchyma. The infection of the glia limitans may explain some reports of cerebellar *T. cruzi* infection (28, 32) as there is a glia-cerebellum neural connection.

## Invasion of neural cells by *T. cruzi*

The neurovascular unit (NVU) comprises BBB-endothelial cells, pericytes, astrocytes, glia, neurons and the extracellular matrix (ECM); all being involved in regulating cerebral blood flow and BBB function. Therefore, any dysregulation of NVU components should alter brain homeostasis, leading to disease (54). In this way, experimental studies have shown that *T. cruzi* infects microglia, macrophages, astrocytes, and neurons (30, 55, 56). Furthermore, in CD patients, the parasite was found in microglia, endothelial cells, and macrophages (18). Also, histological and *in vitro* evidence of *T. cruzi* parasitism revealed high frequency of parasite nests without surrounding membrane in non-glial cells and parasites in astrocyte processes among Purkinje cerebellar cell bodies (28, 49).

Interestingly, astrocytes seem incapable to control *T. cruzi* by the secretion of IL-1β and NO, turning them more permissive to parasite replication than microglial cells (57). In addition, astrocytic processes surrounding the CNS vessels may facilitate parasite invasion, especially in the gray matter where capillary density is higher than in the white matter (28). Some biological processes have been described during CNS *T. cruzi* infection, which could promote parasite replication and tissue damage through the ligation between the parasite-derived trans-sialidase (TS) and the neurotrophin receptors TrkA and TrkC (55, 58), which are commonly upregulated during CNS injury, infections, or degenerative diseases. These interactions facilitate parasite adherence and efficient invasion of neuronal, epithelial, and phagocytic cells *in vitro* and *in vivo* (59); also activating MAP kinase (MAPK) and phosphatidylinositol 3-kinase/Akt signaling pathways, as seen in infected PC12 cell line, an established model of neuronal differentiation (60).

Lastly, systemic or *in situ* pro-inflammatory cytokine release can promote *T. cruzi* invasion. *In vitro* and *in vivo* experiments showed that IFN-γ-expressing glial cells as well as TNF-α levels correlate with astrocyte invasion (49, 56). Also, parasite infection drives astrocytes to a pro-inflammatory profile with enhanced IL-6 and TNF-α production and TNFR1 expression, potentially favoring TNF signaling (49). This suggests a self-sustaining inflammatory loop creating a favorable environment for parasitic replication.

## Oral *T. cruzi* infection: Possible dissemination routes after oral mucosa epithelium traversing

The oral infection in humans is characterized by more severe manifestations than those associated with vectorial transmission: prolonged fever, acute myocarditis, heart failure, and meningoencephalitis (61). At least in Brazil, acute human CD occurs after oral infection, with a potential higher inoculum in patients infected by the oral route, as compared to those infected by the vectorial route (61). Importantly, the presence of *T. cruzi* in various tissues located in the anatomical pathway between the oral mucosa and the CNS has been reported (12, 32, 62). Therefore, in principle, after invading the oral mucosa, parasites might migrate to the blood and/or lymphatic fluids from the submucosa, through the palate and tissues of the nasal cavity. These data unravel the existence of multiple interactions between the *T. cruzi* and the diverse anatomical regions of the oral mucosa (12).

We found that some soft regions of the oral cavity and underlying tissues are invaded by *T. cruzi* in orally infected mice, including the cheek muscle, salivary glands, and submandibular lymph nodes (12). Hypothetically, the lymph node invasion occurs by drainage of infected tissues, implying a route of infection towards more distant tissues such as the heart, liver and spleen following the drainage route.

We also found that the naso-maxillary region, nasal cavities, and subjacent tissues are invaded by *T. cruzi* in orally infected mice. *T. cruzi* DNA was also observed in brain tissues after oral infection (12), suggesting that the parasite could reach the brain *via* invasion of the olfactory nerve and later the olfactory bulb. Corroborating this idea, intranasally *T. cruzi* infected mice showed infection of several brain regions (32), indicating that the oral cavity and the adjacent nasal compartment invasion represent a putative anatomical route for parasite spreading to the brain.

Active *T. cruzi* penetration into the host cell involves first a step of adhesion for later penetration of cells. ECM proteins could be relevant players in early oral mucosal infection. In humans, the major basement membrane components of palatal mucosa are laminin, type IV collagen, and fibronectin (63). Similarly, *T. cruzi* can take advantage of ECM to invade CNS tissues. For instance, fibronectin is present in the meninges and the choroid plexus (27), and together with laminin, composes the blood brain barrier (BBB) related basal lamina surrounding the cerebral endothelial cells (53). Actually, several *T. cruzi* target molecules are ECM components of the oral mucosa and the CNS. *T. cruz*i-derived peptidases, like the Tc80 oligopeptidase B, hydrolyses human type-I and type-IV collagens, as well as fibronectin, which is important for the parasite transit through the ECM (64). Similarly, Tc85 is capable of binding host cell derived molecules, such as fibronectin, laminin and heparin (64). Also, parasite penetrin has affinity for ECM elements such as heparin, heparan sulfate proteoglicans (HSPGs), and collagen, promoting fibroblast adhesion and penetration (65), suggesting a role of *T. cruzi* penetrin and neuronal tissue alterations.

Other molecular group includes the parasite-derived TS family. Inactive forms function as parasite adhesins, binding to sialic acid and β-galactose residues, whereas active TSs transfer sialic acid from host glycoconjugates to acceptor molecules on the parasite surface, regulating the process of cell adhesion, migration, and invasion (66). Strikingly, the expression of active TSs during the trypomastigote life cycle stage seems to favor the epithelial cell invasion (67). Considering that epithelial cells from the oral cavity are enriched in sialic acid, it is conceivable that *T. cruzi* takes advance of sialoglycoproteins, invading the cavity and evade host defense. Interestingly, TSs have also neuraminidase activity and can interact with neurotrophin receptors TrkA and TrkC, promoting both invasion and survival of neurons and glial cells (58, 59).

**Figure 2:**
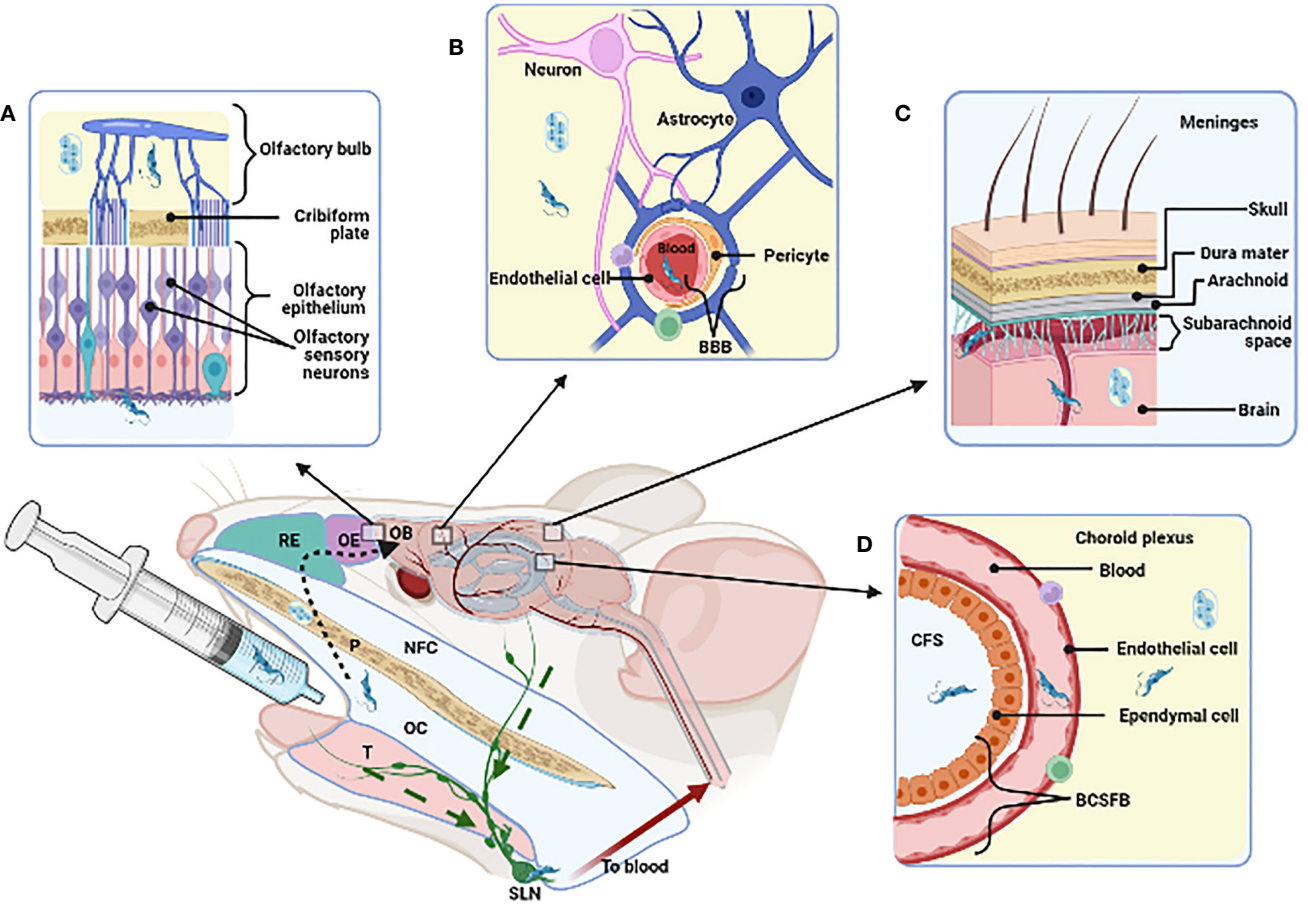
Strategies that *T. cruzi* may use for reaching and invading the Central Nervous System and possible dissemination following oral cavity (OC) infection. The figure stands for murine experimental oral infection. Accordingly, after delivering the parasites into de oral cavity, they may take the following pathway(s): 1) olfactory way (**A**, dotted arrow), traversing the palate (P), nasopharyngeal cavity (NFC), respiratory and olfactory epithelia (RE, OE) reaching the olfactory bulb (OB); 2) disrupture of the blood brain barrier (BBB) in the parenchyma **(B)** or in the meninges **(C)**; 3) disrupture of the cerebrospinal fluid barrier (BCSFB) in the choroid plexus **(D)**; 4) invasion of the submandibular lymph node (SLN) by drainage of infected tissues (green dashed arrow). Tongue (T). Created with BioRender.com.

Whichever route is used to spread from the oral cavity, parasites can ultimately disseminate, crossing the endothelium of both blood and lymphatic vessels, the oral epithelium at different location and the blood-brain barrier (BBB) to invade the brain, as schematically depicted in **Figure 2**.

## Conclusions and remaining questions

The data summarized herein clearly show the CNS commitment in acute and chronic CD. Different cell types in the brain can be infected by *T. cruzi*, including neurons, astrocytes and microglia. Also, various regions of the CNS can be affected, such as choroid plexus, hippocampus, meninges, olfactory bulb, pituitary gland, among others. The cellular mechanisms involved in *T. cruzi* infection in the central nervours tissues has also been largely documented, comprising both parasite-derived and host-derived moieties; among others ECM ligands such as fibronectin, laminin and glycosaminoglycans (summarized in **Table 2**).

**Table 2:** Molecular interactions between *T. cruzi* and host molecules.

*T. cruzi*-derived protein	Interacting host protein	Function	Interacting protein expression in the human mucosa/CNS	References
Glycosylphopsphatidyl-inositol (GPI) molecules	TLR2	Calcium signaling activation - Infection	Human epithelial cells of oral-nasal cavities	(68)
Glycoinositolphospholipid (GIPL) molecules	TLR4	Calcium signaling activation - Infection	Epithelial cells of oral-nasal cavities	(68)
gp82, gp35/50, gp30, and gp90 (GPI)	Gastric mucin	Calcium signaling activation - Infection	Gastric epithelial cells	(69)
Cruzipain	Kininogen	Calcium signaling activation – Infection	CNS	(46)
TGF-β-like molecule	TGF-β receptor	Attachment - Infection	Glia, neuron	(70)
β-galactose glycoconjugates	Galectin 3	Attachment - Infection	Oral epithelial cells; Brain macrophage/microglia	(64, 71, 72)
Trans-sialidase	TRKA	Infection	Neurons, dendritic cells, astrocytes and microglia	(60)
Trans-sialidase	TRKC	Infection	Neurons, dendritic cells	(58)
Tc85	Cytokeratin 18 - Laminin	Adhesion - infection	Oral mucosa; Oral palate; Blood brain barrier	(53, 63, 73)
Penetrin	Heparan sulfateproteoglycan (HSPG)	Adhesion - infection	CNS	(74)
*T. cruzi* DNA	TLR9	Immunostimulation	Oral epitelial cells and tongue	(68, 75)
Tc80 (Oligopeptidase B)	Heparin G proteins - Fibronectin - Human type I and IV collagen	Calcium signaling activation – infection Parasite transit	Human brain microvascular endothelial cells	(63)

The possibility of *T. cruzi* CNS invasion after oral ingestion raises a series of questions that still need to be answered. Which ligands and/or mechanisms does the parasite use to cross the mucosa and reach the brain tissue? Can the immune system adequately respond to parasite infection in the mucosa and brain? Are there differences regarding CNS commitment after oral infection compared to those caused by reactivation due to immunosuppression? Which brain areas and functions are mostly affected after infection? Which are the systemic consequences due to CNS infection by the oral route? Addressing these issues will provide new clues that may allow earlier diagnosis and treatment for oral Chagas Disease.

## Author contributions

JM and YU contributed to the conception and design of the manuscript. YU wrote the first draft and AP, AB and WS wrote sections of the manuscript and made a substantial intellectual contribution to the work. All authors approved it for publication.

## Funding

This work was partially funded by Fiocruz CNPq, CAPEs, FAPERJ (Brazil), PIP CONICET 0715 (Argentina), and Mercosur Fund for structural convergenge (FOCEM, Mercosur). AB and WS are recipients with grants from CNPq, whereas YU received a Post-doctoral Felowship from FAPERJ (Grant E-26/202.139/2020), Brazil. This work was conducted in the frameworks of the National Institute o Science and Technology on Neuroimmunomodulatiom (CNPq), and the Rio de Janeiro Network on Neuroinflammation (FAPERJ).

## Conflict of interest

The authors declare that the research was conducted in the absence of any commercial or financial relationships that could be construed as a potential conflict of interest.

## Publisher’s note

All claims expressed in this article are solely those of the authors and do not necessarily represent those of their affiliated organizations, or those of the publisher, the editors and the reviewers. Any product that may be evaluated in this article, or claim that may be made by its manufacturer, is not guaranteed or endorsed by the publisher.
